# Neglected Tropical Diseases and the Millennium Development Goals-why the "other diseases" matter: reality versus rhetoric

**DOI:** 10.1186/1756-3305-4-234

**Published:** 2011-12-13

**Authors:** David H Molyneux, Mwele N Malecela

**Affiliations:** 1 Centre for Neglected Tropical Diseases, Liverpool School of Tropical Medicine, Pembroke Place, Liverpool, L3 5QA, UK; 2 National Institute For Medical Research, Ocean Road, P.O Box 9653, Dar-es-Salaam, Tanzania

**Keywords:** Neglected Tropical Diseases, Millennium Development Goals, Disease Control, Disease Elimination, Health Systems, Monitoring and Evaluation

## Abstract

Since 2004 there has been an increased recognition of the importance of Neglected Tropical Diseases (NTDs) as impediments to development. These diseases are caused by a variety of infectious agents - viruses, bacteria and parasites - which cause a diversity of clinical conditions throughout the tropics. The World Health Organisation (WHO) has defined seventeen of these conditions as core NTDs. The objectives for the control, elimination or eradication of these conditions have been defined in World Health Assembly resolutions whilst the strategies for the control or elimination of individual diseases have been defined in various WHO documents. Since 2005 there has been a drive for the expanded control of these diseases through an integrated approach of mass drug administration referred to as Preventive Chemotherapy via community-based distribution systems and through schools. This has been made possible by donations from major pharmaceutical companies of quality and efficacious drugs which have a proven track record of safety. As a result of the increased commitment of endemic countries, bilateral donors and non-governmental development organisations, there has been a considerable expansion of mass drug administration. In particular, programmes targeting lymphatic filariasis, onchocerciasis, schistosomiasis, trachoma and soil transmitted helminth infections have expanded to treat 887. 8 million people in 2009. There has been significant progress towards guinea worm eradication, and the control of leprosy and human African trypanosomiasis. This paper responds to what the authors believe are inappropriate criticisms of these programmes and counters accusations of the motives of partners made in recently published papers. We provide a detailed response and update the information on the numbers of global treatments undertaken for NTDs and list the success stories to date.

The paper acknowledges that in undertaking any health programme in environments such as post-conflict countries, there are always challenges. It is also recognised that NTD control must always be undertaken within the health system context. However, it is important to emphasise that the availability of donated drugs, the multiple impact of those drugs, the willingness of countries to undertake their distribution, thereby committing their own resources to the programmes, and the proven beneficial results outweigh the problems which are faced in environments where communities are often beyond the reach of health services. Given the availability of these interventions, their cost effectiveness and the broader development impact we believe it would be unethical not to continue programmes of such long term benefit to the "bottom billion".

## Background

Over recent years, the profile of what are referred to as Neglected Tropical Diseases (NTDs) has increased as a result of several developments including increased advocacy for new approaches to the control or elimination of these diseases, the increased commitment from pharmaceutical donors to provide drugs, renewed government commitment and the recognition that these diseases should be addressed as part of the Millennium Development Goal (MDG) agenda where they are considered as "other diseases" of MDG 6. Significant commitments in bilateral resources have been made by the US and UK governments, whilst the Director-General of the World Health Organization (WHO) has emphasised the need for countries to address these diseases, which afflict poor populations in the world's least developed countries. It is now recognised these diseases are key impediments to development as a result of their health, educational and socioeconomic impact. There are a number of World Health Assembly Resolutions, which mandate WHO and member states to address control and elimination targets for specific neglected tropical diseases. WHO reports on a regular basis to the World Health Assembly on the progress Member States have achieved. The arguments for increased resources to address the NTDs was based on the deliberations of meetings hosted by WHO and Deutsche Gesellschaft fur Technissche (GTZ - now Internationale Zusammenarbeit) in 2003 and 2004 [1,2] with the subsequent publication of a series of papers advocating for an integrated approach to their control [3-6]. In the interim, WHO developed an overarching strategy including guidelines for preventive chemotherapy [7] and published in 2010 its First Global Report on Neglected Tropical Diseases [8] entitled "Working to overcome the global impact of neglected tropical diseases". A summary of the information in the WHO report is available in [9]. Recent peer reviewed publications [10,11] have been critical of various aspects of the momentum to advance the health and well-being of those afflicted with NTDs and have openly criticised the approach to mass drug distribution. These criticisms were based on a limited analysis of case studies in two countries. This paper provides a response to those criticisms as they fail to give adequate attention to many examples of successes in reducing the burden of these diseases in numerous validated and peer-reviewed reports. The papers [10,11] also fail to cite important references relating to monitoring and evaluation, sustainability, drug efficacy studies and health systems research topics, which are the subjects of critical comments.

## Review

### Critical and Sceptical Voices

A recent voice of scepticism of NTD initiatives has been published in *Third World Quarterly*, 32, 91-117 by Tim Allen and Melissa Parker in 2011 entitled "The "Other Diseases" of the Millennium Development Goals: rhetoric and reality of free drug distribution to cure poor's parasites"[10]. The authors [10] misquote the aim of MDG 6 by including TB, which is not specifically included in the formal MDG statement, which reads "to combat HIV/AIDS malaria and other diseases". The paper [10], whilst referring to several pertinent publications, does not cite or comment on papers in a *Lancet *Series on NTDs that offer critical evidence in support of NTD interventions [12-16]. Nor does it refer to a paper [17] published in 2008, which addresses the arguments for investment in NTD control using the title "Other Diseases" and Millennium Development Goals. These authors' non-selection of these references is germane, since they do, in fact, include an editorial comment from the *Lancet *in February 2010 to support their arguments, despite the fact that the commissioned *Lancet *Series on NTDs was published the previous month (January 2010) suggesting these papers were ignored. The Series on NTDs in the *Lancet *was accompanied by a peer-reviewed Comment [12] outlining the arguments for the increased investment in NTD control whilst the front cover page introducing the *Lancet *series headlined the following quote "Only 0.6% of overseas development assistance for health is allocated to neglected tropical diseases affecting at least 1 billion people" thereby emphasising the context. This statement comes from the analysis by Liese and Schubert [18] on the official Overseas Development Assistance for health being committed to NTD control in comparison with other health development activities.

However, having failed to quote the *Lancet *Series the month before, Allen and Parker refer to the *Lancet *editorial of February 2010 [19] and comment as "excoriating" implying "excoriating" refers to NTDs although the editorial was directed at criticism of UNICEF. Allen and Parker [10] state "but identical observations might soon have to be made about the current target obsessed approach to drug distribution for NTD control". These authors make several statements in their paper [10], which need rebuttal in detail, given the available published data. Significant papers and current programmes have not been referred to in any detail nor have the historic and sustained successes of NTD control been recognised. A summary table of NTD programme successes was published [17] and an update is included in this paper.

### Putting progress into context and addressing misconceptions

Statements made by Allen and Parker [10], however, need comment as it is suggested that published data are invalid or inaccurate. There is no doubt that in some settings there are problems with reporting; Allen and Parker are correct to identify such issues, which are common to all health programmes not just those targeting NTDs. However, the impression gained from the paper [10] is that the WHO published figures are inaccurate or incorrect. Phrases such as "it is claimed" that 670 million [8,9] people were treated for NTDs via preventive chemotherapy in 2008 are used in [10] when WHO, in its normative role, has the obligation to provide to the international health community the figures which they are given by Member States. Such comments [10], throw doubt on the integrity of both the endemic countries and WHO. Allen and Parker [10] state that there is a growing body of research that suggests distributing drugs "is facing serious problems". At no time in the paper do the authors refer to the successes of programmes. These NTD successes have been described [3,17]. Allen and Parker [10] do not refer, for example, to the successes, of the African Programme for Onchocerciasis Control (APOC), the Leprosy programme, Trachoma control, in the Guinea Worm Eradication Programme, in human African trypanosomiasis activities and several country successes in lymphatic filariasis. We summarise these achievements at the end of this paper.

### "Active resistance"

Allen and Parker refer to "active resistance" against free drug treatment programmes.

The evidence from over one hundred projects of the African Programme for Onchocerciasis Control (APOC) in 19 African countries, where sustained delivery of ivermectin for the control of onchocerciasis, has been ongoing in hyper- and meso-endemic areas since 1996, is ignored. This programme is now reaching a population of over 68 million with annual treatments and as drug deliverers at the community level are unpaid, this hardly suggests "active resistance" [8]. This position is supported by an APOC evaluation [20] of the sustainability of projects by standard and rigorous evaluation procedures; the conclusions in [20], undertaken in 492 communities representing 41 APOC projects in 10 countries suggest that coverage and compliance was higher when no incentives were given to community distributors. Some 70% of these projects were considered to be satisfactory or highly sustainable, it was recognised that weak health systems that resulted in late delivery of ivermectin could impair sustainability, but there was no "active resistance". A recent paper by Brieger et al. [21] directly addresses the issues raised by Allen and Parker [10] focussing particularly on compliance with community directed mass drug administration (MDA) and relating the interactions between compliance and coverage in a study in five sites in Nigeria and Cameroon involving over 8000 participants. Allen and Parker [10] cite various observations about the resistance to treatment following adverse events in Tanzania and Uganda. In the APOC programme, partners have sought to address the serious adverse events, which occurred in the early part of the programme associated with the use of ivermectin in communities co-endemic with the Tropical Eye Worm, *Loa loa*. In certain areas of Cameroon and Democratic Republic of Congo (DRC) treatment with ivermectin resulted in a *Loa-*induced encephalopathy in approximately 1 in 10,000 treatments [22,23]. APOC, the drug donor, Merck & Co. Inc., and the Mectizan Donation Program immediately took steps to produce guidelines for the use of ivermectin in areas of high risk, initiated research on the alternate approaches and supported the development of mapping tools to define those high risk areas [24,25]. The problems associated with expanding MDA for lymphatic filariasis in areas of potential co-endemicity with *L*. *loa *have delayed implementation in central Africa and the recent WHO Review document and Strategic Plan for lymphatic filariasis 2011-2020 considers addressing the challenge of co-endemic loiasis a priority [26]. This approach reflects the responsibility of those associated with NTD control to concerns over safety with the recognition that continued research is necessary to find optimal solutions. There is a need to reduce the risk of adverse events to be balanced with the broader public health goals of elimination of a public health problem through risk-benefit analyses.

### "Technique Transplantation" - what the evidence actually says

Allen and Parker [10] refer to the quotation from the first Director-General of WHO, Dr Brock Chisholm, regarding "transplanting techniques" and refer to MDA for NTD control. The issue of technique transplantation in reference to the current MDA strategies cannot be classified in such a way. Indeed the whole purpose of the community directed approach developed by APOC [27] is community ownership as the core principle, while MDA for deworming is often led through the education sector as opposed to the health sector, or in emergency and conflict settings through the World Food Programme or Non Governmental Development Organisations (NGDOs). The value of Community Directed Interventions (CDI) for onchocerciasis control has been demonstrated in a multi-country study [28] as a means of implementing other health interventions with particular reference to the increased coverage with bednets for malaria control and the Home based Management of Malaria as well as Vitamin A distribution. The WHO/UNICEF guidelines also provide information on deworming in Vitamin A programmes as a safe and cost saving intervention for children. Homeida et al. [29] identified some 20 additional health activities, which were undertaken by community directed distributors (CDDs) in the APOC programme with no remuneration; for Allen and Parker to suggest that this implies "resistance" to participation in such health programmes is at variance with facts. It is useful to compare the quotes provided by Homeida et al. [29] as they provide contradictory views about community MDA compared with those referred to in [10]. In addition, the distribution of drugs for onchocerciasis and lymphatic filariasis in Nigeria increased the bednet uptake for malaria amongst pregnant women who were excluded from the MDA (because they were pregnant) by nine-fold [30]. The initial studies on the use and potential of community based or directed treatment were assessed in both Africa and India for filariasis drug distribution, but in India it was found that populations preferred to receive treatment from the health services rather than through the communities. This has, however, not reduced the ability of the filariasis programme in India to deliver through the health system 292.8 million treatments in 2010 in 165 implementation units out of a target population of 377 million without any external support [31]. A further factor ignored by Allen and Parker is the extent of deworming activities promoted by the World Bank through the education sector and the role of the Partnership for Child Development in promoting deworming to improve growth and weight gain [32] (including rapid growth spurts), reduced stunting, and increased school attendance (decreasing absenteeism by 25%) and improved cognitive performance scores. These are well-defined outcomes of regular deworming of school age children. Michel and Kremer [33] reported that as a result of school deworming programmes in Kenya, worm infection rates fell by 50%. They identified significant externalities of deworming in school age children summarised in a recent presentation by Bundy [34] which emphasises that deworming through preventive chemotherapy, is an important part of development strategy, a fact identified by the UN Special Advisor to the Secretary-General Jeffrey Sachs in a UN Report identifying "quick wins" towards the MDGs published in 2004/5 [35] - improved physical growth and general health, improved learning abilities and educational opportunities, externalities leading to long term economic benefits demonstrated by several authors in different settings [32-37].

Baird et al. [38] have undertaken a prospective study of deworming carried out in Kenya [33], which began in 1998; they used a new data set which tracked 83% of treated subjects who are now between 19 and 26 years old. Subjects had received two to three more years of deworming than a comparison group. Among those in employment, earnings were 21% to 29% higher in the treated group, hours worked increased by 12%, and work days lost to ill health declined by a third. The earnings gained were explained by shifts, for instance, in a doubling of those in manufacturing employment compared to a reduction of those in casual labour. Those who were self-employed also improved their income significantly. Total years enrolled in school, test scores and self-reported health improved significantly. The authors conclude that deworming has very high social returns, with conservative benefit-cost ratio estimates ranging from 24.7 to 41.6 [38] justifying the conclusions of the UN Millennium Development Goal report [35]. Similarly, an independent assessment of the cost effectiveness of mass chemotherapy interventions for onchocerciasis, soil transmitted helminths (STH) and lymphatic filariasis [39], as part of the Disease Control Priority Project showed that these mass preventive chemotherapy interventions were amongst the best health buys available as measured in terms of Disability Adjusted Life Years (DALYs) averted [39]. As onchocerciasis and lymphatic filariasis interventions are also providing "deworming" drugs the impact on improved health, productivity and societal benefit to the 887.8 million treated people receiving the interventions must be profound [8] if the studies from Kenya are extrapolated globally.

In addition, Bleakely [40] has analysed retrospectively the impact of elimination of hookworm in the southern states of the US, which started in 1910 supported by the Rockefeller Sanitary Commission. At that time, some 40% of children were infected with hookworm. Treatment and education campaigns rapidly reduced the disease. Areas with higher levels of hookworm infection prior to the campaign experienced greater increases in school enrolment, attendance, and literacy after the intervention. No significant contemporaneous results are found for literacy or occupational shifts among adults, who had negligible prior infection rates. A long-term follow-up indicates a substantial gain in income that coincided with hookworm elimination. There was also evidence that the return to schooling increased with elimination [40].

After accusations of "technique transplantation," Allen and Parker then state with reference to the "target obsessed approach" to NTD control that "Monitoring and Evaluation are systematically inadequate". This appears to be a non-sequitor. Target obsession would surely be related to monitoring and evaluation. The most recent overview of monitoring and evaluation by Baker et al. [15] is not quoted by Allen and Parker; neither do they refer to the detailed data produced by WHO in the Weekly Epidemiological Record (WER) for all the NTDs in a systematic way each year or to peer reviewed publications on several programmes. All up-to-date country data are available on line at WHO NTD sites referring specifically to each NTD disease on an annual basis, for example [31,41,42] with information on target numbers and reported coverage where appropriate. In the case of Guinea Worm (Dracunculiasis) the numbers of reported cases in the remaining endemic countries are published on a monthly basis with comparisons with data from previous years. In addition, reports from Guinea Worm countries in the state of pre-certification that have not reported a case for over the last 12 months are also included [42]. This documented information is in the public domain and derived from endemic countries. Other information is available in peer reviewed journals. To suggest, therefore, as Allen and Parker do, that "there is much more going on at the moment than the straightforward presentation of evidence about NTD control" is not only an inappropriate assertion but also not in accordance with the facts. The statement suggests the data are "overblown assertions". Ignoring the use of the word "claim" in [10], WHO in its latest update published in October 2011 reported global treatments for onchocerciasis (68.7 million), lymphatic filariasis (485.2 million), soil transmitted helminths (314 million) and schistosomiasis (19.9 million); hence in total 887.8 million people received Preventive Chemotherapy [9,43] for elimination and control of these four NTDs. The figure for soil transmitted helminths includes data reported through school-based programmes and from the World Food Programme (WFP), Save the Children, World Concern, Helen Keller International (HKI) and other NGOs, and there may be overlapping treatments when individuals receive treatments for more than one disease in a reporting year. On the basis of these figures, at least one tenth of the planet is receiving drugs for NTDs. This, in itself, whatever the opinions of Allen and Parker, is a massive achievement by endemic countries and their diverse partners. To suggest the figures are "overblown assertions" calls into the question the veracity of many organisations as well as the national authorities involved. The criticisms regarding this form of delivery by Allen and Parker fail to recognise the numbers of deworming treatments and the value of these activities in educational terms [33,37]. There are few reports that this regular intervention has evoked the emotive stories or active resistance suggested by Allen and Parker (page 109).

### What is the real burden of NTDs? or anything else!

Allen and Parker consider that the numbers of people infected with NTDs and the Disability Adjusted Life Years (DALY) burden calculated and attributable to NTDs "can be little more than guesstimates". That assertion could apply to most of the DALY estimates for almost all diseases and conditions. The NTDs are caused by a spectrum of different biological agents-viruses to worms and the resulting clinical conditions are equally diverse, some being rapidly and inevitably fatal if untreated, others causing life-long disablement. However, from the perspective of DALY burden, the critical issue is the value of disability weights attributed to these diseases and their true prevalence. In addition some NTDs cause cancers, (food- borne trematodes and schistosomiasis), epilepsy (cysticercosis) or injuries (by dog bites caused by rabies) and such outcomes are not attributed to NTDs [44]. The current estimates of NTD annual mortality is circa 500,000 [5,6,8]. Whilst this figure is an estimate, it must be borne in mind that NTDs, as causes of death in rural settings in the developing world, are dependent on quality reporting from peripheral health units where diagnosis, even if a death is reported, is unlikely to be attributed to an NTD as reporting forms do not necessarily identify particular NTDs; this particularly applies to the zoonotic NTDs or Neglected Zoonotic Diseases [44]. What can be said is that in terms of annual mortality NTDs are broadly the same as the estimated numbers of maternal deaths, reducing maternal mortality being a specific MDG.

### Impact of MDA via Preventive Chemotherapy on clinical disease

In reference to the reported studies undertaken by Allen and Parker in Tanzania [10] regarding the use of ivermectin and albendazole on the impact on clinical disease, they state that the drugs "did not appear to have an impact" on cases of hydrocele and lymphoedema. In some cases patients are indeed in an advanced clinical state, precluding any chemotherapeutic or surgical intervention which would impact on their condition. However, the authors [10] do not record the sample size of the people interviewed as there are several published reports in Tanga and Morogoro, Tanzania of post MDA improvement in clinical condition-reduction of filarial fevers or homa za mitoki [45,46]

However, the Global Programme to Eliminate Lymphatic Filariasis (GPELF) [31] is designed as a preventive chemotherapy programme in parallel with a lymphoedema management programme. In Tanzania a President's Fund has been established to enable patients to access hydrocele surgery. Morbidity programmes have often lagged behind the MDA as a component of the GPELF. However, there is strong evidence that the drugs used in the LF programme do reduce the frequency of filarial fevers (dermato-lymphangioadenitis), and, if given early enough, "are capable of reversing the sub-clinical lymphatic damage in children and provide other benefits other than the interruption of transmission" [47], whilst in Papua New Guinea [48] state "Mass drug administration...results in immediate health benefits by decreasing the incidence of acute attacks of leg and arm in people with pre-existing infection." They state, "Results of our study indicate that mass distribution of anti-filarial drugs has immediate clinical benefit to endemic communities in addition to future transmission cessation". These studies directly contradict the assertions of Allen and Parker that clinical benefits do not accrue from MDA whilst there is proven reversal of pathology in schistosomiasis in 90% of patients treated with a single dose of praziquantel after 6 months [49,50] and a reduction in itching (pruritus) after treatment with ivermectin [8,21].

### Addressing criticisms of countries - a case of neocolonialism?

For its evidence, the study [10] focuses on case studies in two African countries. The major concern here is that the methodology in some of the areas is flawed but nevertheless has resulted in the authors drawing generalized conclusions about country NTD programmes. It is important that the criticisms leveled at countries are also addressed.

For example, the authors have focused on Tanzania as one of their case studies but we express considerable concern on the methodology used, particularly in the LF programme, where no attempts were made to gather information at the regional and national levels. It is interesting to note that the region which contains districts that Allen and Parker [10] describe as their study sites in Tanzania is one that has a robust monitoring system for LF and which, since 2004, has been monitoring infection in humans as well as infection and infectivity in mosquitoes. There have also been coverage surveys that have been reported, but no reference to these is made whilst [10] emphasizes that there is no monitoring and evaluation (M & E) of these programmes. The published study from Kiarare [51], the sentinel site for the Tanga region was not referenced. This is all the more surprising given Kairare in Pangani district is one of the sites quoted by Allen and Parker [10].

Allen and Parker quote that "In Tanzania there is a persistent practice of increasing treatment numbers as reporting was passed up the system". This is an assertion that needs to be supported with evidence and using the data at village, ward, district, regional and national levels. There is no reference to these additional levels in the Allen and Parker text. It is important to stress this because the paper makes reference to exaggerated figures but does not look at the whole structure of the health reporting system and the checks and balances which are embedded at each level. Data from Tanga region in Tanzania do not show the 100% coverage that is referred to, and information from the monitoring site in Kiarare shows clearly that the coverage is not and never has been 100% [51]. If the researchers had probed further they would have noted that no regional LF coordinator would accept a figure of 100% and that such a reported result would have been referred back for verification.

Allen and Parker go to some lengths to explain how people call elephantiasis by different names and hence cannot make the linkage between the disease, its vector and eventually the MDA. The issue of nomenclature is something that is well known and earlier studies by Gasarasi et al. [52] have shown this. Had the authors critically reviewed the IEC material written in Kiswahili, focusing on the local terms for the disease, they would have noted that this was a fact that was taken into consideration in the development of these IEC materials. Further south they would have encountered several different names for the same conditions. It is important to state that Allen and Parker worked in a district of 44,107 people (Pangani) and 279,423 (Muheza); from this experience they inferred their results were applicable to the whole country. There is no mention of the limitations of such inferences and no mention of the ecological and social heterogeneity of Tanzania which the authors are fully aware of.

Of serious concern, however, are the factual errors which call into question the credibility of the authors and the case studies which are published to support the authors' claims [10]. The work in Tanzania refers to an integrated programme which was not in place until 2009. The references to an NTD programme in Tanzania are erroneous. The reference to the riots in Morogoro being the reason for the slow pace of the NTD programme is not supported by the facts. Negotiations for programme implementation began in 2004. There was a consensus building process that involved the lymphatic filariasis, onchocerciasis, schistosomiasis, soil transmitted helminth and the trachoma programmes. The authors should have made reference to the process that led to the formation of the integrated NTD programme in 2009.

The paper [10] combines events in Tanzania and Uganda. For example, the statement "drugs are distributed by district vector control officers". This is not the case in Tanzania where the District LF coordinators and more recently NTD coordinators are involved in the delivery of the drugs. Issues are lumped in a manner that suggests what is happening in Uganda is considered to be the same in Tanzania. Such comments allow generalizations that are over- simplistic and fail to take into account country structural differences.

However, we recognize that Allen and Parker [10] (pages 101-102) do make valid comments and raise pertinent questions, which need to be addressed by NTD programmes. These include; 1) what proportion of children living in endemic areas attend school when school based programmes are used?, 2) what proportion of children who do not attend school receive drugs?, 3) what proportion of adults receive drugs from MDA?, 4) how are these free drugs perceived by adults and by children?, 5) are they being swallowed?, 6) do local understandings of the disease affect consumption?, 7) are there any indications that individuals are becoming more aware of the benefits of treatment? The answers to several of these questions have indeed been addressed in attempts to determine the target populations and assess the total coverage of the population. The groups of people who are not eligible suggest that overall coverage of between 65-75% is an acceptable level. Reported coverage is backed up in some programmes by spot checks referred to as surveyed coverage. The availability of free drugs and the perception of benefit are borne out by the sustained levels of coverage on the one hand [20,21,31] whilst the actual swallowing of tablets is assured by a directly observed treatment approach in some national programmes. As far as awareness of benefit is concerned, the sustainability studies and the recognition of clinical benefit have been recorded in [20,21,45-48].

### Inappropriate tone of criticism

Several authors appear to have induced exasperation by "grandstanding rhetoric" and "exaggerated claims". This paper seeks to refute such inappropriate comments in a respected journal. The suggestion that "achievements" are "purported" is a serious allegation which requires to be challenged in the public domain. Those individuals and organisations, including the Director-General of WHO, have been misrepresented in motive and accused of the use and presentation of selected data. There is overwhelming evidence of massive achievements, which have produced documented health and economic benefits, strong country commitment, pharmaceutical donations of billions of treatments and proven successes [8,9,12,17]. It is indeed time to separate rhetoric from reality. The paper [10] is highly subjective, using examples from limited field studies and failing to quote or deliberately ignoring the body of literature, which supports the arguments for NTD control as a massive contribution to the health of the poorest. NTD interventions are highly cost effective and cost beneficial interventions [16,39] - it is indeed time to examine reality.

### Drug resistance and/or reduced efficacy

Reality is not served by ignoring the issues of potential loss of efficacy or drug resistance; nor is the need for new products for those diseases where, for many decades, tools have been inadequate such as human African trypanosomiasis, Chagas' disease and leishmaniasis. The need for effective diagnostics as well as tools for monitoring and surveillance remains for many NTDs. Indeed organisations such as the Drugs for Neglected Diseases Initiative (DNDi) and, the Foundation for Innovative Diagnostics (FIND) have been established to address these issues. WHO in response to concerns about potential drug resistance from albendazole, commissioned an independent multi-country study to assess the efficacy of the drug against hookworm and other intestinal parasites [53] and found no evidence of lack of efficacy in hookworm. The NTD community is profoundly aware of the need for vigilance. However, to suggest that the NTD community is unaware of these needs is incorrect. The research plans for individual programmes have been published, for example [26,54], and the deficiencies in our research knowledge emphasised by WHO [8]

The Allen and Parker paper refers to the Financial Times letter of Professor Bruno Gryseels. His warning that there are risks of drug resistance are recognised, as WHO has undertaken and supported efficacy studies on albendazole [53] and it is recognised that there is some evidence of loss of efficacy of ivermectin in some populations in Ghana [55]. However, this is in a restricted area and alternative approaches such as doxycycline antibiotics targeting the *Wolbachia *endo-symbiont bacteria could be deployed in these areas and elsewhere [56,57]. Gryseels et al. [58] warned of the risk of praziquantel resistance in an outbreak of *Schistosoma mansoni *in Senegal which resulted in the drug being used less than the public health situation demanded. Fenwick et al. [59] review the evidence for resistance to praziquantel in different settings and, while there is evidence of loss of efficacy in some studies, it appears that true genetic resistance has not been detected. The suggestion that praziquantel is withheld as a public health tool on the basis of possible resistance is not an acceptable position.

### NTDs and the Health System

In a recent paper, Cavalli and colleagues [11] described the interactions between the Neglected Tropical Disease Control Programme in Mali and the Health System. The paper makes the valid point that Global Health Initiatives (GHIs), of which there have been many emerging over the past decade [60], have the potential for distortion of the overall needs of the health of the population and detract from the provision of the necessary care and, in the process, "burden" the health system. It is appropriate to respond to various points raised in the light of the policy debate and the review of the Millennium Development Goals in relation to diseases of poverty and the health MDGs generally.

In criticising the Mali NTD programme and being necessarily selective in the regions and districts studied the paper [11] analyses the first year of activities of the NTD programme. It is well known that any project has up-front higher start-up costs than occur in subsequent years. What is not recognised is that the training of trainers has a multiplier effect in energising health care 'beyond the end of the road' in areas where populations frequently have no access to any government service; it seems unethical to allow communities to remain disenfranchised from any health care if donated products are available with wide spectrum efficacy and can be delivered at low cost [16]. Health care must start somewhere and, as APOC has demonstrated, when some 50% of the treatments of ivermectin are distributed to communities more than 20 km from any health facility [61] the argument that NTD programmes do not strengthen but detract from the development of the health systems fails completely. If there is no health system it is impossible to strengthen it. Recent APOC reports suggest that not only are APOC projects sustainable [20,21] but that the numbers of health workers and community-directed distributors who have been trained exceeds 450,000 [61] a significant contribution to strengthening health systems. Cavalli et al. [11] fail to recognise that disease control activities are an integral part of the health system and should never be separated from it as the proponents of the Cavalli et al. view seem to believe [58]. This argument applies to every programme where the target is an infectious agent, as the overall objective of any programme for infectious disease control, elimination or eradication, is to reduce incidence, be it through vaccination, mass drug distribution, vector control or case finding and treatment. The objectives of disease control activities must also be seen in the light of biological feasibility as well as in a public health and policy context. Authors critical of the preventive chemotherapy approach [10,11,58] seem not to recognise that many millions of people in Africa (and in Asia and Latin America) have benefitted and will continue to benefit from NTD programmes. The argument of Cavalli et al [11] is that these programmes damage the health system. The latest figures for Africa show that 68 million are treated annually for onchocerciasis [61] (2010), 82 million for lymphatic filariasis in 2010 [30] in 19 endemic countries and 37 million for trachoma in 19 countries, the majority being in Africa [62]http://www.who.int/blindness/publications/GET15REPORT4.pdf. The drugs used for preventive chemotherapy of onchocerciasis and lymphatic filariasis also benefit populations because of the synergistic impact on intestinal worms [4,12,17]. These programmes have the objective of reduction of morbidity and thereby the prevention of blindness or long-term disability. The fundamental question for those that suggest that preventive chemotherapy programmes should await the strengthening of health systems or resources deployed to strengthen such systems is simple. Would they countenance and take responsibility for allowing the risk of blindness and disability to continue when free drugs are available which are effective and prevent long term sequelae of intense infections? Such a position is unethical and counter to the Right to Health and the WHO Charter. Strengthening health systems is a laudable objective but a long-term one. In the meanwhile, MDA can be delivered by the communities themselves or through schools at costs that have been assessed to be in cents rather than dollars per person treated per year [see summary of papers in [16]. Indeed annual deworming in Asia has been costed at around US$ 0.02-0.04 [63]. The cost effectiveness of the lymphatic filariasis programme, annual deworming and the onchocerciasis programmes based on ivermectin have been independently assessed to be amongst the most cost effective public health buys as a part of the extensive Disease Control Priorities Project [39]. If we fail to deliver what we have now and await the development of stronger health systems then many poor people will become blind, stigmatised and disabled both physically and mentally. Nor are we aware of the impact of NTDs on the mental health of sufferers and their carers. This remains to be quantified in terms either of numbers or burden. The issue of the mental health burden of NTDs together with neglected zoonotic diseases, (some of which are included by WHO in its categorisation of NTDs), are two examples of outstanding issues on the NTD agenda to be addressed [44], and add a further dimension of neglect and underestimated burden.

## Conclusion

Allen and Parker imply the advocates of picking what has been described as "low hanging fruit" [17] are somehow misguided, have questionable motives and misrepresent results. These are assertions we address by providing evidence from the peer reviewed literature, from historic successes of NTD control strategies when they are applied, sometimes at a vast scale and provide references to counter incorrect and invalid assertions. We also refute specific criticisms from the country perspective. There is no doubt that there remains much to be addressed and a series of papers commissioned by the WHO Special Programme for Research and Training on Tropical Diseases (TDR) point out the issues relating to the social science research needs [64]. Parker and Allen provide a further critique of the NTD programmes in Uganda [65]. Justifiably there will be ongoing debate on the issues surrounding all aspects of NTDs from a policy context to the scientific priorities for individual diseases. However, it is inappropriate to question the motives and the integrity of individuals on the one hand and to challenge the right of national governments to act as they wish on the other. Our position is clear - it would be unethical not to distribute to as many as possible, donated, quality, safe (albeit within limits specified by the manufacturers and with ongoing monitoring) and efficacious drugs with a broad impact on the lives of poor people. These drugs prevent disablement, reduce the risk of progression to irreversible disability (skin disease, blindness, gross lymphoedema, hydrocele and bladder cancer), and can reverse symptoms of early pathology (e.g. anterior segment eye pathology and initial trachoma lesions, lymphatic damage and associated fevers). They also enhance educational performance, improve female and maternal health and birth outcomes [6,8] and potentially reduce risks of HIV transmission [66]. This indeed is "low hanging fruit" which it is appropriate to pick; to reject this position seems unethical and grossly negligent. Similarly we refute the views of Cavalli et al. [11] who consider that NTD programmes detract from the priority, which they perceive should be the strengthening of health systems. Cavalli et al [11] consider that NTDs, as well as other vertical programmes, distract health staff in systems with limited resources from the routine responsibilities of care. However, an opposite view is that such programmes strengthen systems in a number of ways, are ethically appropriate given the role of communities in distribution, the role of community distributors in providing other health interventions [28,29], the limited access that such communities have to the health system (if they have access at all) and an obligation to provide products, which prevent disability on a huge scale. To take a contrary view is counter to the "Right to Health". We would ask a simple question in response to Cavalli et al [11] as the paper is based in Mali; would it be acceptable NOT to distribute ivermectin or azithromycin to communities where there was a severe risk of blindness when free drugs and other resources were available, which if they were not given would commit many thousands of people, including children, to a life of misery - in the case of onchocerciasis progression to severe itching, to irreversible posterior segment ocular lesions with resulting blindness, which could never be resolved even in the most sophisticated health system!! In the case of trachoma it would commit children to the pain and distress of trichiasis and the need for later surgery at high cost. Prevention of such tragedies is the only alternative when there is no cure and entirely in accord with the principles of primary health care.

Allen and Parker [10] focus on the major policy debate at a global level but are disrespectful to endemic countries. The problems of under and over reporting are issues that countries are tackling everyday; a problem not only for NTD programmes. Obtaining good data from any field programme is a problem, which no one disputes; hence the application of spot check surveys to ascertain the reported surveyed coverage. No country, would accept that their system is perfect but should countries stop such NTD programmes until such data systems are robust? Is it fair or ethical that such cheap and effective interventions are denied the poor when delivery is possible at grassroots level and beyond existing health services? The tone of the paper [10] is contradictory. The fact that it questions the motives of the international communities and partnerships, authors and advocates as well as the endemic countries, which hitherto have not prioritized these diseases is not only damaging but irresponsible; worst it is damaging to poor people who benefit. The further contradiction is the accusation that NTD proponents are "target obsessed" and then accuse NTD programmes of inadequate monitoring evaluation, which is illogical. More constructive and appropriate responses to the issues confronting NTD control and health systems derive from papers by Marchal et al. [67] and Utzinger et al. [68] where a more balanced and measured assessment of the programmatic complexities are analysed. However, NTD programmes seem to be the target of such analyses and are often considered as some kind of "parallel" system despite countries themselves considering them to be a totally appropriate activity for a health system and a core part of their function. This position is anomalous when equal and indeed even stronger criticism can be levelled at the Global Fund for AIDS, TB and malaria and the polio eradication programme. These programmes which have also been accused of distorting country priorities [17] do so to an extent far greater than any NTD programme given the volume of resources committed to the "big three" [17].

We consider that we cannot wait for health systems to improve. Indeed NTD control provides the very platform on which they can be built [69]. This paper rejects and refutes the sentiments expressed by Allen and Parker and Cavalli et al. [11] and provides detailed responses to their assertions and claims by referencing peer reviewed publications on NTDs not referenced by them and providing references to data from many countries where NTD control is a part of country health programmes. The section below provides a summary of some of that information.

### Selected successes in the Control of Neglected Tropical Diseases

The Allen and Parker paper [10] on page 94 asks two questions; "Can they [NTDs] be so readily controlled and what has been achieved so far? Our answer to the latter two questions is that, perhaps not surprisingly, things are very much more complicated than is claimed". Below are documented accounts of the successes of NTD programmes, which refute the suggestion that NTDs cannot be readily controlled.

**Lymphatic Filariasis **has been successfully controlled in China in a population of 350 million people and in the Republic of Korea (South Korea); transmission has also been arrested in several countries where it is no longer a public health problem [54]. WHO [31] has reviewed the status of 9 countries originally classified as endemic but found not to require MDA (Burundi, Cape Verde, Costa Rica, Mauritius, Rwanda, Seychelles, Solomon Islands, Suriname, and Trinidad and Tobago) as there was no evidence of transmission [31]. However, Egypt [70], Togo, Yemen, Cambodia, Vietnam, Maldives, Sri Lanka and 7 Pacific Island nations who implemented MDA early in the programme have reduced transmission and met the criteria of stopping MDA. These countries require transmission assessment surveys as recommended by WHO. WHO has reported that around 500 million treatments are being distributed each year [26] with savings of US$ 24 billion between 2000-2008 [71]. Annual treatments of ivermectin and albendazole are given in Africa where onchocerciasis is co-endemic. In the rest of the world the drugs used are diethylcarbamazine (DEC) and albendazole. Ivermectin and albendazole also have a significant impact against intestinal worms. As a result of the programme up to 2008, 66 million newborns have been prevented from becoming infected, 2.2 million protected from developing clinical disease and 28.7 million who have problems of existing infection have seen their clinical symptoms diminish and not progress to further disability. The most recent data from WHO [31] reported that by the end of 2010, 53 countries had implemented drug distribution programmes of the 72 now recognised endemic countries. Country data reported to WHO for 2010 showed that 622 million people had been targeted for MDA and 466 million had been treated giving a reported coverage of 75%. However, several countries are yet to initiate MDA in Africa.

**River Blindness (onchocerciasis) **has been eliminated as a public health problem and as a disease of socio-economic importance in 10 West Africa countries, the original area of the Onchocerciasis Control Programme (OCP) protecting a population of some 50 million people; the benefits of the OCP have been quantified as 600,000 cases of blindness prevented, 18 million born free of the risk of blindness, 25 million hectares of arable land reclaimed for settlement and agricultural production. This programme which started in 1974 and continued with uninterrupted donor support until 2002 has been widely recognised as one of the most successful health and development programmes ever executed both in terms of health and development gains but in terms of World Bank investment [72]. Control of blindness and skin disease via the donated drug ivermectin (Mectizan; donated by Merck & Co. Inc) is now reaching over 68 million people each year in 17 countries by the APOC supported by national governments and Non Governmental Development Organisations through over 748,000 community workers trained in 120,000 communities since 1995. In Africa there is evidence that 15-17 years annual distribution of ivermectin has eliminated transmission in Mali and Senegal [73] providing strong evidence that elimination is possible with ivermectin alone. Onchocerciasis is also endemic in 6 countries in Latin America where twice yearly distribution of ivermectin has arrested transmission in 4 foci. in Colombia, Guatemala and Mexico and interrupted transmission in a further 6 other foci [74].

Domestic transmission of **Chagas disease **due to *Trypanosoma cruzi *has been controlled in five South American countries by domestic spraying of insecticide against the vector *Triatoma infestans*, providing economic rates of return of around 30% on the investment in vector control. In Central America, progress has been reported through control of *Rhodnius prolixus*. Transmission by blood transfusion has been substantially reduced throughout Latin America. Sustaining the advances made and maintaining an effective surveillance system are necessary whilst research for new and effective drugs continues to be a high priority to treat those infected.

**Leprosy **has been reduced as a public health problem as a result of the use of multidrug therapy of three donated drugs- rifampicin, dapsone and clofazimine. Of the 122 countries considered endemic for leprosy, WHO states that 119 have eliminated the disease as a public health problem (defined as 1 case per/10,000). The 213,000 cases reported are confined to 17 countries reporting more than a 1000 cases/year. The figures suggest a reduction of 90% in endemic countries through case finding and multidrug therapy, which have prevented disabilities in between 1 and 2 million people. Since 1985 some 14.5 million people have been cured through multidrug therapy. The numbers of new cases per year have fallen dramatically [8,41].

**Guinea Worm **is moving towards eradication. The numbers of cases have been dramatically reduced from over 1 million in 1988 to 1797 in 2010 [42]; countries with ongoing indigenous transmission are Chad, Ethiopia, Mali and Sudan. There are several countries, which have not reported cases during the previous year (Burkina Faso, Cote d'Ivoire, Ghana, Kenya, Niger, Nigeria, Togo) and are considered to be in the pre-certification phase awaiting formal certification as being free of transmission. Post-certification, there is a continued need for surveillance until global eradication is declared. The Weekly Epidemiological Record (WER) of WHO provides monthly reports on data from the remaining endemic countries and those yet to be certified as free of transmission - the pre-certification countries. The latest WER reporting all the country data reported to WHO from 2010 can be found in [42].

**Schistosomiasis **affects some 200 million people. Intensive control in Egypt has reduced prevalence from around 20% to less than 1-2% using the drug praziquantel (now 0.32 US$/treatment) over the last two decades of both *S. mansoni *and *S. haematobium *[59]. Schistosomiasis transmission in Egypt has been largely eliminated over the last five years and control focuses on hotspots of transmission and the result has been a massive reduction in incidence of bladder cancer. China has also made considerable progress and now there are less than 1 million people reported to be infected [75]. Programmes in Africa are now reaching school age children in 17 countries in Africa and initial results show that dramatic reduction in prevalence over a period of 4 years of annual treatment.

A **Trachoma **programme has been established to eliminate blinding trachoma by 2020 through the SAFE strategy (S = surgery; A = antibiotics; F = facial cleanliness through washing; E = environmental control). Trachoma is endemic in 57 countries and the cost of the disease in terms of lost productivity is estimated at US$ 2.9-5.3 billion/annum. The antibiotic azithromycin (Zithromax) is donated. Three countries have reported reaching their ultimate intervention goal targets (Iran, Morocco and Oman). There is a need for further upscaling in the highest burden countries such as Ethiopia, Nigeria and Sudan. There were 37 million treatments of donated zithromax in 19 countries in 2010 [62].

**Human African Sleeping Sickness**. Over the period 1999-2009 the numbers of reported new cases of both *Trypanosoma brucei rhodesiense *and *T. b. gambiense *sleeping sickness has declined by 65%, the numbers of new cases reported falling from over 28,481 to 9,878. However, these figures are likely to be underestimates because of the remoteness of many endemic areas that may not be covered by regular surveillance. There is evidence that the disease is no longer present in many West African countries probably due to climate change and population pressure on habitat of the tsetse fly vector, *Glossina*. The problem remains focussed in Central Africa. In 2009 only 2 countries reported over 1,000 cases - DRC and Central African Republic followed by Chad (510 cases), Sudan (376) and Angola (247) - of *T. b. gambiense*; more extensive surveillance and the availability of treatment provided through WHO of donated drugs is the likely cause of the reduced incidence reported. There remains a need to maintain effective surveillance in historic foci and provide diagnostic tests. There has also been a reported decline in cases of acute *T. rhodesiense *of 58% in East and Southern Africa 1999-2009, from 619 to 190, a 70% decrease http://www.who.int/gho/neglected_diseases/human_african_trypanosomiasis/en/index.html. The adoption of a cattle treatment and insecticide spraying of cattle as a strategy to reduce the reservoir of human infective parasites in Uganda has had a major impact on transmission of *T. rhodesiense *to humans [76].

**Soil transmitted helminth **control targets three nematode worms, which inhabit the gut; hookworm (*Necator *and *Ancylostoma*), whipworm (*Trichuris*) and roundworm (*Ascaris*) and whose global prevalence is probably greater than all the other NTDs combined. Some 882 million children are estimated by WHO [77] to need preventive chemotherapy (273 million pre school age and 609 million school age). WHO reported that 109.7 million pre-school children (proportion of total 33.7%) and 204 million school age (proportion 29.9%) were treated. The overall coverage around 30% is below the global target number treated, which was 313.7 million in 2009, an increase of over 100 million since 2008. Annual mass drug distribution of the drugs mebendazole or albendazole through deworming programmes usually by school-based delivery have a significant impact on educational achievement, increased growth and weight gain, cognitive and physical performance [32,36,37]. Deworming of pregnant women in the second and third trimester of pregnancy increased child survival at the age of 6 months by over 40% in areas of hookworm endemicity. The costs of these deworming programmes in South East Asia are of the order of 2 US cents/year [63]. The onchocerciasis and lymphatic filariasis programmes also act as deworming programmes as the drugs used have powerful effects on the worms of the gut.

## Competing interests

David Molyneux is, in part, supported by a grant to the Liverpool School of Tropical Medicine, Centre for Neglected Tropical Diseases, from the UK Department for International Development and GlaxoSmithKline.

## Authors' contributions

DHM wrote the initial draft. MM contributed specific information about the Tanzania NTD activities. Both authors read and approved the final manuscript
